# Defining Clinical Trial Estimands: A Practical Guide for Study Teams with Examples Based on a Psychiatric Disorder

**DOI:** 10.1007/s43441-023-00524-2

**Published:** 2023-05-27

**Authors:** Elena Polverejan, Michael O’Kelly, Nanco Hefting, Jonathan D. Norton, Pilar Lim, Marc K. Walton

**Affiliations:** 1 Statistics and Decision Sciences, Janssen Pharmaceuticals - Johnson & Johnson, 1125 Trenton-Harbourton Rd, Titusville, NJ 08560 USA; 2 Center for Statistics in Drug Development, IQVIA, Dublin 3, Ireland; 3 Global Clinical Development, Therapeutic Area Psychiatry, H. Lundbeck A/S, Valby, Denmark; 4 Statistical & Quantitative Sciences, Takeda Pharmaceuticals U.S.A., Inc., Lexington, MA USA; 5 Quantitative Sciences Consulting, Statistics and Decision Sciences, Janssen Pharmaceuticals - Johnson & Johnson, Titusville, NJ USA

**Keywords:** ICH E9(R1), Treatment effect, Intercurrent events, Missing data, Stakeholder, Estimator, Depression, Major depressive disorder

## Abstract

While the ICH E9(R1) Addendum on “Estimands and Sensitivity Analysis in Clinical Trials” was released in late 2019, the widespread implementation of defining and reporting estimands across clinical trials is still in progress and the engagement of non-statistical functions in this process is also in progress. Case studies are sought after, especially those with documented clinical and regulatory feedback. This paper describes an interdisciplinary process for implementing the estimand framework, devised by the Estimands and Missing Data Working Group (a group with clinical, statistical, and regulatory representation) of the International Society for CNS Clinical Trials and Methodology. This process is illustrated by specific examples using various types of hypothetical trials evaluating a treatment for major depressive disorder. Each of the estimand examples follows the same template and features all steps of the proposed process, including identifying the trial stakeholder(s), the decisions they need to make about the investigated treatment in their specific role and the questions that would support their decision making. Each of the five strategies for handling intercurrent events are addressed in at least one example; the featured endpoints are also diverse, including continuous, binary and time to event. Several examples are presented that include specifications for a potential trial design, key trial implementation elements needed to address the estimand, and main and sensitivity estimator specifications. Ultimately this paper highlights the need to incorporate multi-disciplinary collaborations into implementing the ICH E9(R1) framework.

## Introduction

Clinical trials were traditionally planned as follows: a general trial objective was stated, then the trial design, analysis sets, and statistical methods determined how the treatment effect was estimated. This approach was not optimal, because the definition of what was being estimated by the trial was either not stated clearly or not stated at all.

The ICH E9(R1) Addendum [1] on “Estimands and Sensitivity Analysis in Clinical Trials”, released in 2019, (hereafter referred to as “the Addendum”) recommends a change in the process of planning, design, conduct and reporting of clinical trials. The Addendum emphasizes that to properly inform decision-making by various stakeholders and to provide clear descriptions of benefits and risks of a treatment, it is important to have precise descriptions of the treatment effects of interest reflecting clinical questions posed by trial objectives (i.e., the estimands) that are clearly understood and relevant to support the decision(s) to be made by the stakeholders. Estimands must be documented in the protocol; trial design and all aspects of trial conduct and the planned analyses flow from their specification. As pragmatic considerations may impinge on the feasibility of estimating a specified estimand, this process will, in practice, be iterative.

The Estimands and Missing Data Working Group of the International Society for CNS Clinical Trials and Methodology (ISCTM Estimand WG) includes members representing both clinical and statistical functions, with both trial and regulatory experience. This working group had the objective to develop an interdisciplinary process for implementing the estimand framework in the planning stage of a clinical trial. The current paper describes such a process, illustrated by specific examples using hypothetical trials evaluating a treatment for major depressive disorder (MDD). The description of this process and the examples are intended to be a practical aid to clinical trial teams in applying the recommendations of the Addendum to clinical trials across many disease areas.

Section "Process for Selecting and Constructing Estimands" of this paper describes the recommended process for selecting and constructing estimands and highlights key points regarding the estimand attributes. Section "Process for Selecting an Estimator Aligned with an Estimand" describes the process of selecting an estimator aligned with an estimand. Section "Estimand Examples for Major Depressive Disorder" presents multiple examples of estimands for MDD, some with examples of aligned estimators. Section "Discussion" includes discussion points and further thoughts on this topic.

## Process for Selecting and Constructing Estimands

As noted in the Addendum, the purpose of a study is to support decision-making by one or more stakeholders who will use the study results. The precise question(s) each stakeholder needs to answer to support their decision-making can be different, and thus different estimands could be defined for each stakeholder identified for a trial.

The ISCTM Estimand WG recommends the following steps in applying the estimand framework:

- Identify stakeholder(s)
- State decision(s) to be made by each stakeholder
- Define objective(s)
- Under each objective supporting main decision making:
  - Formulate the clinical question of interest:
    - Consider the clinical context
    - Consider potential intercurrent events (ICEs) and how they relate to the question
  - Define the corresponding estimand
  - Justify the utility of the selected question and corresponding estimand to the specific stakeholder(s).

This process may, in practice, be iterative. If an estimand is determined not to be estimable, a relevant alternative question of interest that is aligned with the selected objective should be sought.

### Identify Stakeholder(s) and Decision(s) to be Made

There are often a variety of stakeholders who will make decisions based on the results of a clinical trial. Health authority agencies (HAAs, such as FDA, EMA, Health Canada, PMDA etc.) might for example need to decide whether a study contributes substantial evidence of short-term efficacy for a new treatment or that a new treatment is effective as maintenance treatment after an initial short-term response. A company developing a new drug might for example need to determine whether a study provides enough evidence of efficacy to decide on continuing its development. Payers might need to determine whether a study contributes substantial evidence of clinically meaningful patient-level benefit for a new drug or whether the decision to prescribe a new drug is more clinically effective over a long-term period than the decision to prescribe another well-established drug. Eventually payers make decisions on whether to include a drug in a formulary, and what level of payment to provide in relation to available products. Physicians and patients will need enough information to enable their individual decision-making on starting a treatment. This might include answering the questions: what benefit can be expected in patients who could adhere to treatment? How likely is it that the treatment would be adhered to?

Estimand examples in "Estimand Examples for Major Depressive Disorder" section highlight the variety of stakeholders for a study and the decisions they need to make. While these examples highlight decisions on the efficacy of a new treatment, such decisions are complemented in practice by those based on safety and risk–benefit evaluations.

### Define an Objective(s)

Each objective should support the stakeholder’s decision making. For example, if the decision for a HAA is to determine if the study contributes substantial evidence of efficacy for a new monotherapy drug for MDD, the following objective supports this decision (see Estimand 1 example in "Estimand Examples for Major Depressive Disorder" section): *To assess the superiority of new drug versus placebo in short-term symptom reduction when given as monotherapy treatment in MDD patients*. The statistical hypotheses for an endpoint (e.g., superiority or non-inferiority) or the statistical decision rules (e.g., Go/No Go decision rules) relate to the chosen objectives. A trial objective should mention both the treatment conditions that are being compared and the target population for treatment, both being attributes of an estimand (as discussed in "Define the Estimand" section).

Multiple objectives typically inform each stakeholder’s decision making. Protocol templates [2, 3] require that the included objectives reference all endpoints selected for the trial. These objectives are usually prioritized for the trial as primary, key secondary, other secondary or exploratory to distinguish those used for main decisions (primary and key secondary), and those that have supportive or other roles. This distinction is especially important in the regulatory setting. Of note, it is possible for multiple objectives to reference the same endpoint (e.g., for different target populations).

### Formulate the Clinical Question of Interest, Define the Corresponding Estimand, and Justify Their Utility to the Stakeholder

As mentioned above, an objective is a general statement of what supports a stakeholder’s decision. The clinical question of interest is a meaningful and concise definition of the treatment effect, best formulated using natural, non-technical language for easy comprehension; it is paired with a formal, detailed definition of the corresponding estimand. They must be relevant to the stakeholder and have their utility justified. All the estimand examples from "Estimand Examples for Major Depressive Disorder" section include these three components.

#### Formulate the Clinical Question of Interest

The formulation of the clinical question of interest must consider the clinical context of use. This involves consideration of:

- Target population (including typical comorbidities and behaviors)
- Treatment and comparators pertinent to that context and population (including the availability and effectiveness of alternative treatments in the target population)
- Outcome of interest, reflecting the qualitative aspect of the treatment effect (e.g., achieving or avoiding a certain discrete outcome such as treatment success or failure, time to an outcome, change in a continuous score) as well as its temporal aspect (e.g., effect at a fixed time point, over a fixed period, at a variable point in time, over a variable period).

When these have been carefully specified, potential intercurrent events (ICEs) can be considered. ICEs [1] are defined as events occurring after treatment initiation that affect either the interpretation or the existence of the measurements associated with the clinical question of interest (e.g. treatment discontinuation, starting alternative treatments, death; see Sect. Identify ICEs). Once the ICEs pertinent to the clinical context are identified, a study team can formulate a precise clinical question of interest, for example *“For a patient with MDD, what would be the expected effect of prescribing drug X on depression severity at Week 8, were no other antidepressant medications available?”* While this target treatment effect will be formalized in the estimand definition, formulating the clinical question of interest is an important step as it allows a cross-disciplinary discussion in the study team.

The clinical question of interest formulation needs to capture a clear, specific treatment effect of interest relative to each group of identified ICEs. When the estimand is defined (see Sect. "Define the Estimand"), estimand attributes including the strategies selected for the identified ICEs (see Sect. ICE-Handling Strategies, Table 1) will be linked to the clinical question of interest. Examples of types of clinical question of interest formulations (implying different ICE strategies) are presented below:

- Treatment effect *under the assignment to either experimental treatment or placebo***,** *regardless of ICE*—**Treatment policy** strategy
- Treatment effect under a counterfactual scenario (e.g., *as if patients would continue treatment as assigned* or *as if patients would not start other pharmacological treatments for MDD as they were not available)*—**Hypothetical** strategy
- Treatment effect on the *likelihood of a patient experiencing a treatment response*, where the response definition incorporates the ICE (e.g., patient with ICE is considered as non-responder)—**Composite Variable** strategy
- Treatment effect *while treatment is being taken*—**While on treatment** strategy
- Treatment effect in a stratum of patients who would/would not experience the ICE (e.g., in *MDD patients who would adhere to drug X as prescribed for Y weeks*)—**Principal Stratum** strategy.

The examples above are not exhaustive; other language and formulations that link to different ICE strategies could also be used in the question of interest.

The question should be formulated concisely as possible to serve as a guide for the specification of the estimand. Therefore, when formulating the clinical question of interest, some attributes of the corresponding estimand need not be detailed (e.g., exact endpoint, such as the method/scale of capturing depression severity, or exact population-level summary) or may be implied by the description of the effect (e.g., “expected effect” may imply that the population-level summary will be a difference of means).

#### Define the Estimand

The estimand is a formal, operationalized expression of the clinical question of interest, constructed with the following attributes (see Section A.3.3 of the Addendum):

- **Treatment condition of interest and Alternative treatment condition** The interventions being compared. Here, not only the experimental treatment (versus control, if applicable) should be specified but the planned treatment regimen as a whole, including (if applicable) the recommended use of additional or background treatment and/or the strategies for handling ICEs related to the treatment regimen.
- **Population** The population targeted by the clinical question of interest. (It can also reflect a population defined by membership in a principal stratum—see Table 1 for definition of the Principal Stratum strategy). This differs from the analysis set (e.g., all randomized participants), referred to in the past as the analysis population, which should be described under the estimator specifications.
- **Variable (or endpoint)** A value that can be measured in individual patients that is required to address the clinical question, e.g., change from baseline to time X in a measure, time to an event, a binary responder variable. It cannot be a proportion, for example, as this cannot be measured per patient. It can take into account ICEs if the Composite Variable strategy is used, or it can reflect the patient-dependent treatment duration if the While on Treatment strategy is used.
- **Population-level summary** The population-level quantity (derived from the patient-level Variable) that provides a basis for comparisons between treatment conditions and quantifies the treatment effect.
- **ICEs and corresponding strategies** Here, strictly speaking, only the ICEs not covered in the other attributes should be specified together with the strategies used to handle them. However, to improve clarity in this implementation phase, we prefer to list all ICEs and corresponding strategies, including those reflected in other estimand attributes. Patients could experience overlapping ICEs and, if these ICEs are addressed with different strategies, the priority order of applying these strategies must be specified. This will depend on the clinical context; for example, the composite variable strategy will most likely have a higher priority over strategies such as treatment policy or hypothetical (see Sect. ICE-Handling Strategies).

The Addendum recommends at a minimum that estimands for all trial objectives that are likely to support regulatory decisions (such as those related to primary and key secondary endpoints) be defined and specified explicitly. If the trial is to serve multiple stakeholders with different questions of interest, estimands for each stakeholder should be formulated in the protocol or in other prospectively written associated documents. A particular estimand might be of interest to multiple stakeholders, as reflected in some of the estimand examples from "Estimand Examples for Major Depressive Disorder" section.

The following sub-sections provide additional details on the identification of ICEs and on the types of available strategies for addressing ICEs.

### Identify ICEs

All foreseeable ICEs that are likely to be relevant for a trial are to be identified when planning the trial (see Section A.3.1. of the Addendum). The applicable ICEs depend on the specific setting of the trial, but the following is a list of ICEs that are often encountered based on authors’ experience:

- ICEs related to the study treatment:
  - Treatment discontinuation (Tx DC)
  - Change in planned dosage or frequency of administration
  - Treatment non-adherence (i.e., intermittent or partial adherence)
- ICEs related to initiation, adjustment or discontinuation of treatments that are concomitantly taken with the study treatment and may influence the outcome of interest
- Changes in how the outcome of interest is measured (e.g., use of uncertified rater or scale, switching to remote assessment)
- ICEs precluding the existence of values after the event, such as death.

Events could also occur that impact the validity or interpretability of the outcome measurement tool. For example, a cerebrovascular accident could reduce the reliability of assessment of psychomotor impairments attributable to a major depressive episode.

Disease specific regulatory guidance documents for Industry have started to recommend ICEs of interest and strategies to address them, such as the FDA guidance [4] for Chronic Rhinosinusitis with Nasal Polyps or the EMA Guideline [5] on the clinical investigation of medicines for the treatment of Alzheimer’s disease.

On rare occasions a major unforeseen source of ICEs may occur. For example, at the time of writing, clinical trials are being impacted by the COVID-19 pandemic and by the war in Ukraine, resulting in disruption to the provision of drugs, changes to methods of assessment, but also affecting the health of the study subjects, and leading to changes in circumstances (individual or societal) affecting the relationship between disease severity and impairment of function or the reliability or validity of measures designed for use under normal social conditions. In these situations, protocols and other study documents such as Statistical Analysis Plans (SAPs) must be amended to address these unforeseen, major, broadly occurring ICEs [6–9].

Each type of ICE could be considered as a unified event or could be further divided into sub-categories. For example, Tx DC due to different reasons (e.g., due to adverse events, lack of efficacy, or other reasons, such as site closures or other administrative reasons) could be considered as one or as different ICEs depending on reason for Tx DC; likewise different severities of the same event such as low/moderate versus severe treatment non-adherence could be considered separately. Different strategies could then be used if these different events are addressed differently in the clinical question of interest.

ICEs are not synonymous with missing data. Indeed, it is usually desirable to collect data after ICEs, and there are data that are missing without (known) occurrence of ICEs. Study withdrawal is not considered by the Addendum as an ICE. Rather, it is a study event leading to *missing data* (i.e., data that would be meaningful for the analysis of a given estimand but were not collected). Some ICEs might be immediately followed by missing data (which could also be intermittent), while others not. The ICE of death cannot lead to missing data as no measurements exist and can be collected after death.

### ICE-Handling Strategies

ICEs can be addressed by several potential strategies that are described in Section A.3.2. of the Addendum. Table 1 describes each of the five strategies, points to consider on the use of each strategy, and additional considerations on estimation (see Sect. Process for Selecting an Estimator Aligned with an Estimand on the process for selecting an estimator aligned with an estimand). The formulation of the clinical question of interest should drive the selection of strategies addressing the identified ICEs. This requires a collaborative effort across disciplines and is not an exercise for statisticians only.

**Table 1:** ICH E9(R1) strategies of addressing an intercurrent event

Strategy, as described in the Addendum	Points to consider on selecting the strategy	Considerations on estimation aligned to the strategy
*Treatment policy* ICE is considered irrelevant in defining the treatment effect of interest; outcome values are used regardless of whether the ICE occurs	This strategy corresponds to a target effect that could be considered most aligned to the effect of treatment assignment (i.e., being prescribed a treatment), but this does not take into account differences between real-world clinical setting and clinical trials If the ICE is related to treatment (such as Tx DC), this strategy definition can be reflected in the Treatment attribute of the estimand (e.g., as in Estimand 1 example) Suggested description of this strategy in the estimand definition: *Strategy targeting the effect of treatment assignment, regardless of the occurrence of this ICE* Becomes meaningless for terminal events, such as death, which might be handled with other strategies such as the Composite Variable strategy	Strategy requires measurements to be collected post-ICE and included in analysis Nonetheless, there will still be missing data in almost any trial and assumptions on the missingness mechanism will need to be made. In general, applying analysis methods for this strategy based on the Missing at Random assumption without accounting for the occurrence of the ICEs expected to change the patient outcome trajectory (such as treatment discontinuation) could lead to bias [10−12]
*Hypothetical* A scenario is envisaged in which the ICE would not occur; outcome value is that which the variable would have taken in (that) scenario	This strategy could be useful for ICEs that are not considered part of the treatment of interest. An example is when the target effect of assignment to the MDD experimental drug versus placebo does not aim to include the effect of “starting other pharmacological treatments for MDD” in the context of a drug developed as monotherapy treatment The envisaged hypothetical scenario needs to be clearly described in the estimand definition, not left *as if ICE would not occur*. Estimand 1 example description of this strategy is: *A scenario is envisaged in which the event would not have occurred because other pharmacological treatments for MDD are not available.* Estimand 2 example description is: *A scenario is envisaged in which patients would continue treatment as assigned (rather than starting other pharmacological treatments for MDD).* The Addendum mentions that: “A wide variety of hypothetical scenarios can be envisaged, but some scenarios are likely to be of more clinical or regulatory interest than others.” The relevance of each proposed hypothetical scenario needs to be justified If the ICE is related to treatment, this strategy definition can be incorporated in the Treatment attribute of the estimand (e.g., as in Estimand 1 example)	Description of the hypothetical scenario could lead to different assumptions, which could lead to different aligned estimators (see Estimand 1 and 2 examples) When this strategy is used, in most cases data after the ICE is not considered useful in estimation and is therefore not used in analysis. There are also methods that apply adjustments to post-ICE data to model the patient trajectory under the envisaged hypothetical scenario [13, 14] An estimator under the Missing at Random (MAR) such as in example Estimand 2 might be considered to target an effect under optimal conditions (i.e. as if there were no ICEs) and may over-estimate effectiveness in clinical practice
*Composite Variable* The ICE is considered to be informative and is incorporated into the definition of the variable	This strategy reflects the belief that the ICE itself has a (negative or positive) clinical meaning, such as non-response or relapse (e.g., see Estimands 3 and 5 examples)	Direct incorporation of ICE occurrence in the variable through dichotomization tends to involve loss of information. This may be attenuated by ranking strategies [15], assigning unfavorable values to a continuous outcome [16] or by using post-occurrence multiply-imputed outcome from a suitable alternative distribution [17] Incorporating diverse components may make results difficult to interpret; this might require supplementary analyses exploring effects on composite endpoint components
*While on treatment* Outcomes prior to the ICE are of interest	This strategy is applicable to ICEs related to the discontinuation or deviation from the treatment regimen of interest. In the estimand definition, this strategy is reflected in the Variable attribute. Since only pre-ICE measurements are of interest, the Variable cannot relate to a fixed time point, as the duration of study treatment is patient dependent Examples of suitable variables include the area under the curve (see Estimand 7a example), average or a slope derived from pre-ICE measurements or the last measurement prior to or including the ICE occurrence time. This variable must be considered meaningful in the clinical context	This strategy reduces the amount of missing data as the variable is mostly defined based on observed data [18]
*Principal stratum* The target population is the principal stratum in which an ICE would/would not occur under a certain (set of) treatment assignment(s)	In the estimand definition, this strategy is reflected in the Population attribute. Estimand 4 example has the following definition for Population: *Stratum of patients with a diagnosis of MDD, who would adhere to drug X for 8 weeks (i.e., would comply with, complete the treatment, and would not start other pharmacological treatments for MDD by Week 8, if given drug X)* Could take account of the intercurrent event of death by, e.g., targeting the stratum that would not die while on study, irrespective of randomized treatment group As membership in a stratum cannot be observed for all subjects, supplementary analyses estimating probability of belonging to the stratum should also be provided [19]	Membership in a stratum cannot be directly observed for all study participants (e.g., it cannot be observed in the placebo participants in the Estimand 4 example). Modelling assumptions will be required and sometimes research and experience are insufficient. If predictors of membership of the stratum are omitted, the model for membership of the stratum may be inadequate or biased. Standard model checking measures, such as receiver operating characteristic curves and plots of residuals and predicted values, should be presented to allow the reader of the study report to assess the credibility of the prediction of membership of the stratum

## Process for Selecting an Estimator Aligned with an Estimand

For each of the estimands, an aligned method of analysis, or estimator [1], should be implemented that is able to provide an estimate on which reliable interpretation can be based.

Once an estimand is defined and the aligned estimator is selected with the chosen assumptions, the following elements are recommended to be included in the estimator specification:

- Define the estimand and estimator aligned analysis set, specifying not only what trial participants are included (e.g., all randomized) but the selection of measurements to be used for each participant.
  Here, specify what data are not used or missing or sometimes not existing, including:
  • Data not used—Data that may be collected but are not used for the estimator chosen for this estimand, for example the endpoint values collected after an ICE and replaced by imputation;
  • Missing data—Data that would have been useful but could not be collected (e.g., due to withdrawal from the study or intermittent missing)—considered the “true” missing data by the Addendum;
  • Data not existing—such as data after death or, for Principal Stratum estimators, data on the occurrence of ICEs had the patient been assigned to other treatment instead.

- Specify the main estimator for this estimand, including:
  • Assumptions for data not used and missing data; these assumptions, whether the data is treated as missing due to an ICE or simply missing because not collected, inform the scenarios analyzed by the statistical model, and may for example lead to censoring, imputation or generation of a composite outcome.
  • Statistical model and its assumptions (e.g. proportional hazard assumption for Cox regression).

- Specify the sensitivity estimator(s) for this estimand, ensuring that the same estimand is targeted and stating how elements and assumptions differ from those of the main estimator.

Extensive details on selecting estimators aligned with an estimand are provided in Mallinckrodt et al. [20]. Of note, as this is a rapidly evolving field, it is likely that any recommendations beyond those of principle could be superseded. Mitroiu et al. [21] provided a summary of what analysis methods have been commonly used in short-term depression studies, mapping estimands to these methods.

The main estimator produces an estimate for the estimand population-level summary, a clinically understandable estimate of the amount of clinical benefit (or risk, for a safety variable) that was associated with the treatment. This is often loosely referred to as the ‘study result’. As mentioned in Section "Define an Objective(s)", an objective often includes the statistical hypotheses for an endpoint (e.g., superiority or non-inferiority) or the statistical decision rules. Ideally, the analysis used for decision making should be same as the main estimator or at least with similar assumptions. However, it is possible for the analysis used for decision making to be different than the main estimator, especially for the binary and time to event endpoints. As an example, the population-level summary of hazard ratio for a time to event endpoint can estimate the amount of benefit and be derived from the Cox proportional hazard model and the decision-making of superiority can be based on the p-value from the log-rank test. Further research [22–24] is currently being done on constructing time to event methods that could be used for both the main estimator and decision-making.

Section "Estimand Examples for Major Depressive Disorder" includes several examples of estimator specifications.

## Estimand Examples for Major Depressive Disorder

The ISCTM Estimand WG chose MDD to exemplify the process to select and construct estimand, knowing that:

- It is highly prevalent [25, 26] and extensively studied, with widely accepted endpoints.
- Nevertheless, it is a complex indication to pursue, with many challenges, including high treatment dropout rates.
- Many issues encountered in defining estimands in clinical trials of treatment for MDD can be generalized and applied to clinical trials in many other disease areas. These issues include a relatively high number of discontinuations from treatment, (partial) compliance, and starting other pharmacological treatments for MDD that could influence the trial outcomes.

MDD is defined in the Diagnostic and Statistical Manual of Mental Disorders, 5th Edition (DSM-5-TR) [27], by the occurrence of one or more major depressive episodes. Such episodes must be of at least 2 weeks duration, with at least five of nine specified symptoms co-occurring during that period, not attributable to other causes, and leading to impairment of function compared to a state prior to symptom onset. These episodes comprise a primary symptom of subjective or observed persistence and prevalence of either (1) depressed mood (i.e., sad, empty, or hopeless) or (2) markedly diminished interest or pleasure in almost all activities, and additional potential symptoms of (3) spontaneous loss of appetite or weight, (4) insomnia or hypersomnia, (5) fatigue, (6) observable psychomotor retardation or agitation, (7) impairment in ability to think, concentrate, or make decisions, (8) inappropriate feelings of worthlessness or guilt, and (9) recurrent thoughts of death, particularly suicide.

The symptomatic presentations and durations of episodes, and presence, frequency, and patterns of recurrence, as well as level of subsyndromal inter-episodic symptoms are all highly variable both between and within individuals. Thus, pertinent features of MDD as a clinical entity that may impact the choice of estimand in a clinical trial are:

- No single common pathophysiology—samples may comprise pathophysiologic subpopulations that inform patient strata.
- Episodes may be characterized by multiple symptom dimensions [28]—outcome measures must be appropriately responsive to differential treatment effects on symptom dimensions.
- Typical symptoms may differ depending on patient age (e.g., more negative valence system symptoms in younger adults, more prominent positive valence system deficits in older adults) [28]—such differences may inform selection of outcome measures and characterization of patient strata.
- Episodes can have gradual or abrupt onset and offset and duration ranges widely from a defined minimum of 2 weeks, to over a year [29]—consideration of such features is important for time-based elements of study endpoints.
- Episode duration may also differ depending on patient age [30].
- Episode recurrence rates are variable [29]—consideration of such features is important for time-based elements of study endpoints and relevant ICEs.

For the evaluation of monotherapy treatment, short-term, placebo-controlled trials with or without an active reference arm are the usual standard. The short-term, acute treatment trials are typically followed by long-term, randomized withdrawal trials. Drugs may also be developed to be used as adjunctive treatments to existing antidepressant therapy. The MDD estimand examples in this section are presented in the following type of context:

- Short-term monotherapy MDD treatment
- Maintenance monotherapy MDD treatment
- Short-term adjunctive MDD treatment
- Maintenance adjunctive treatment in patients with treatment resistant MDD (TRD).

The MDD examples included in this section follow the estimand framework steps recommended in Section "Process for Selecting and Constructing Estimands". Some of the examples include specifications for a potential trial design, key trial implementation elements needed to address the estimand, and main and sensitivity estimator specifications that include the elements recommended in Sect. Process for Selecting an Estimator Aligned with an Estimand. It is important to emphasize that the presented estimand and estimator examples are not to be taken as guidance; estimand attributes could be described differently and some of the included elements are subject to further research, especially in the field of aligning estimand and estimators. Each of the five strategies for handling ICEs is addressed in at least one example; all examples are considered to be applicable to MDD, based on the authors’ experience.


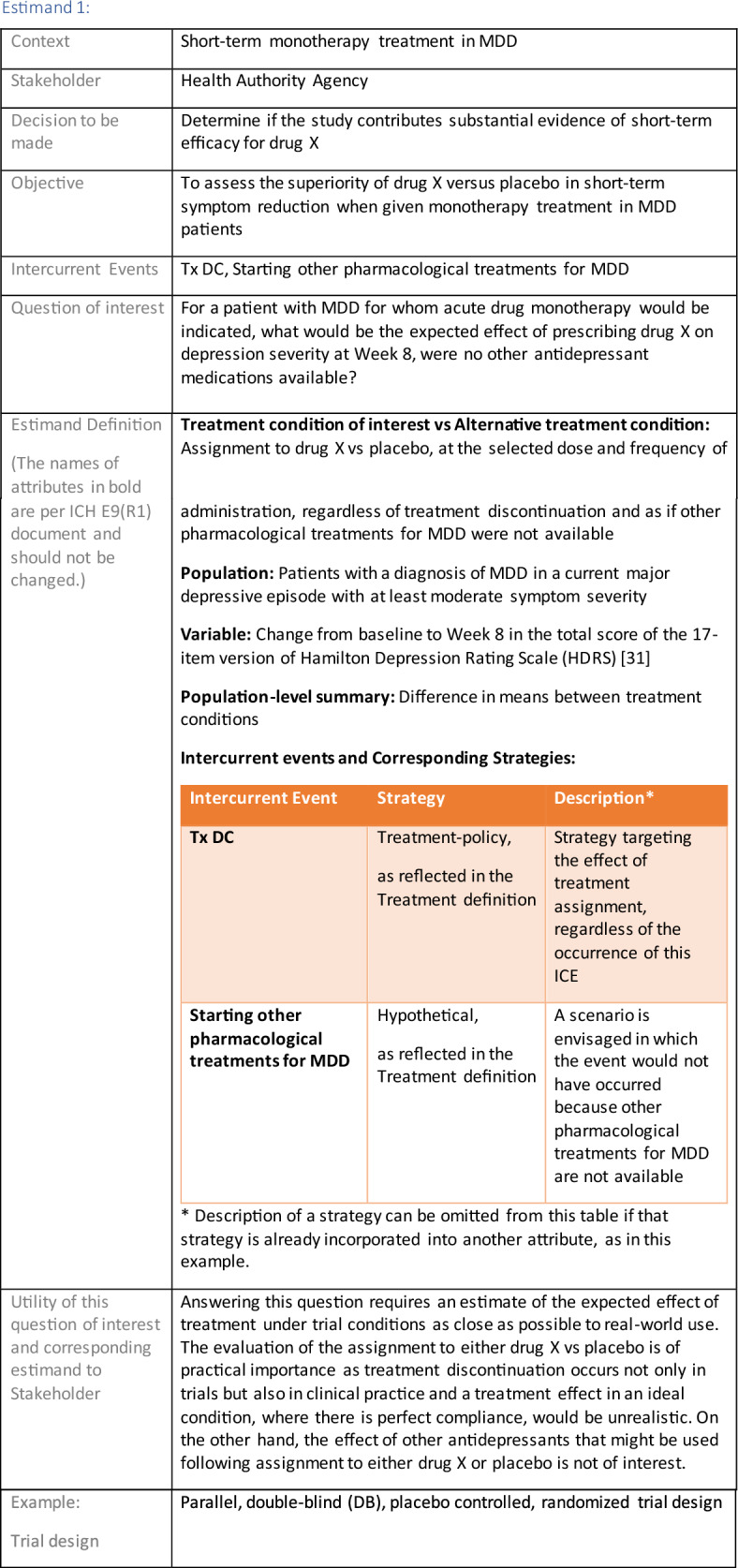
 
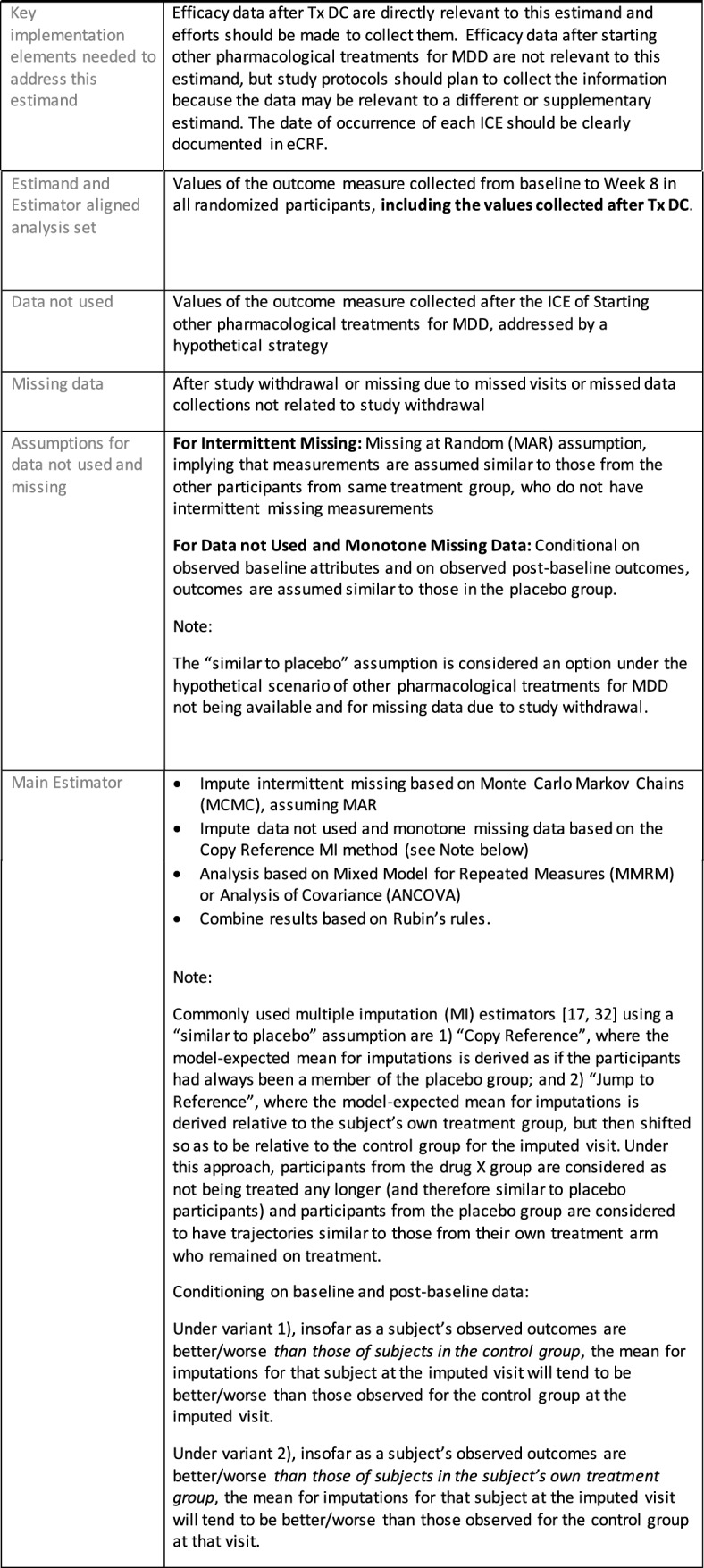
 
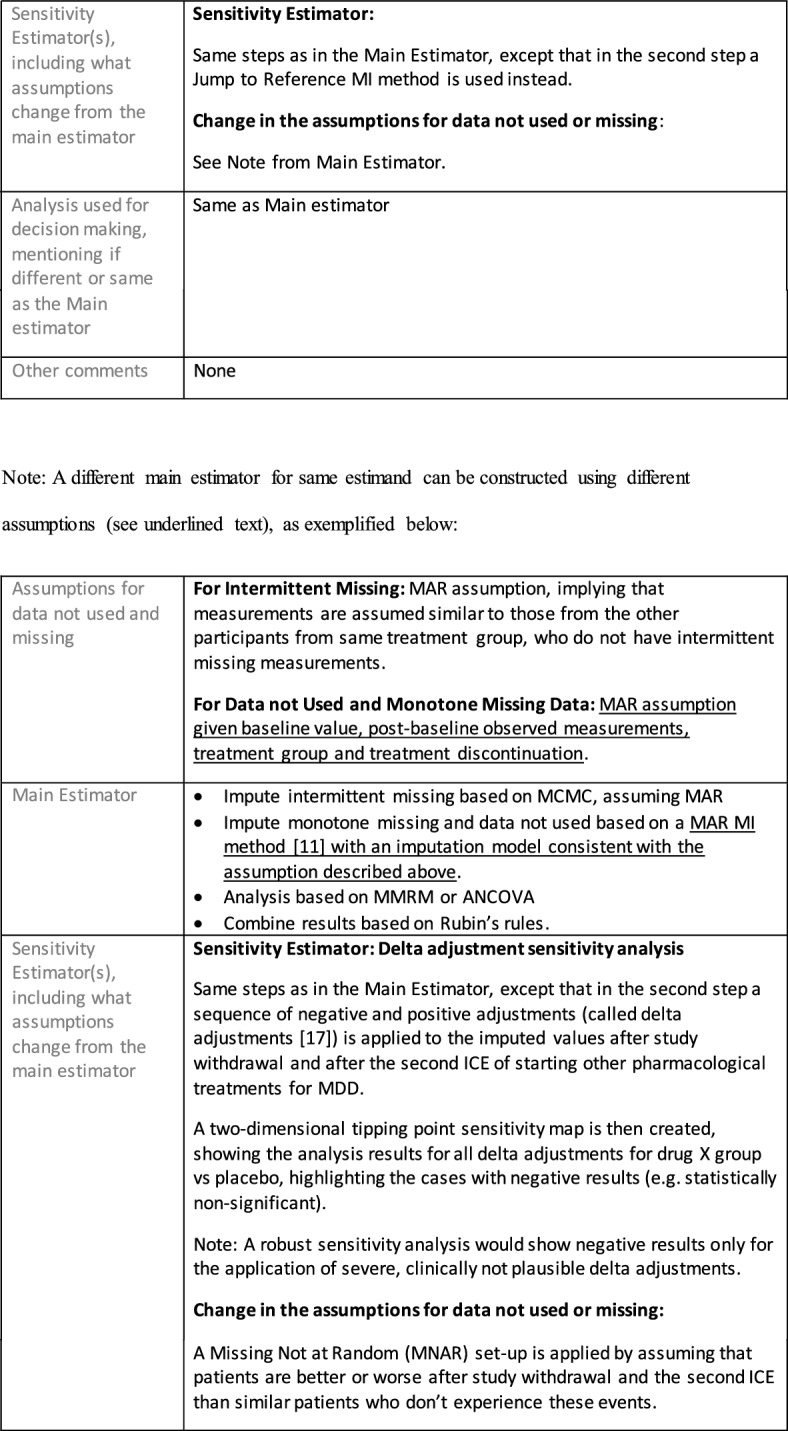
 
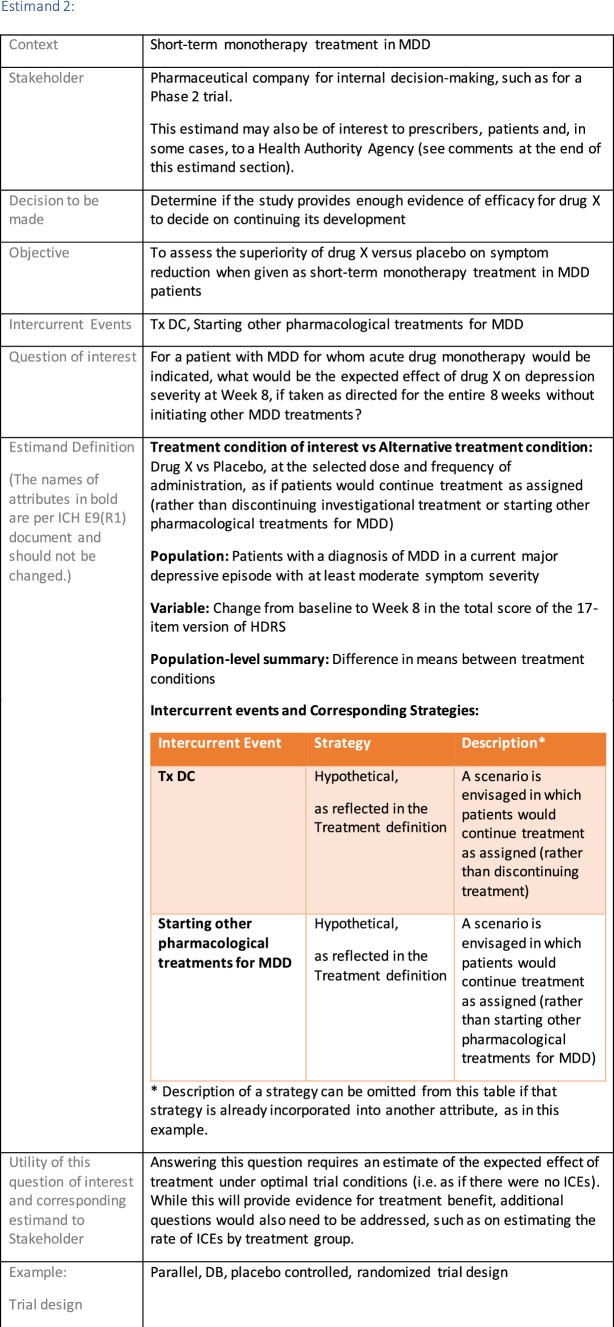



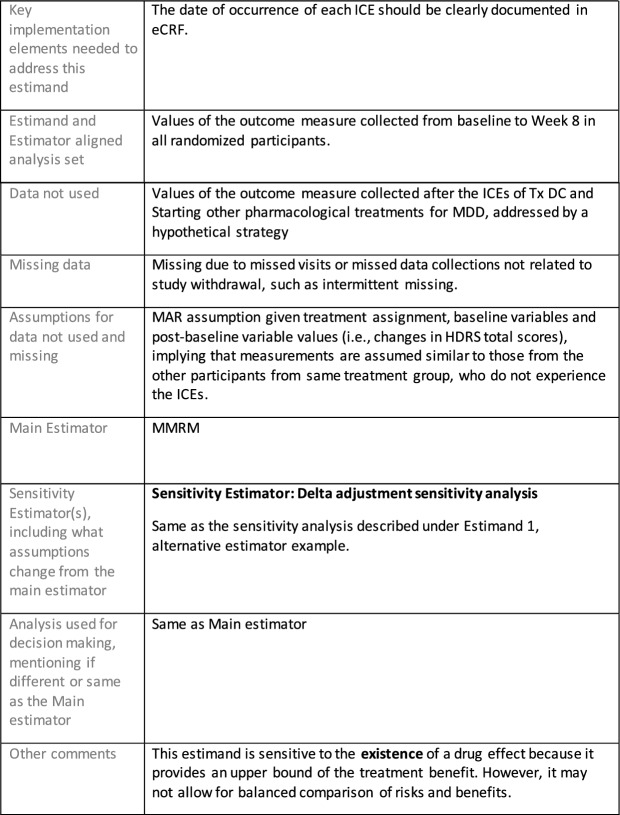
 
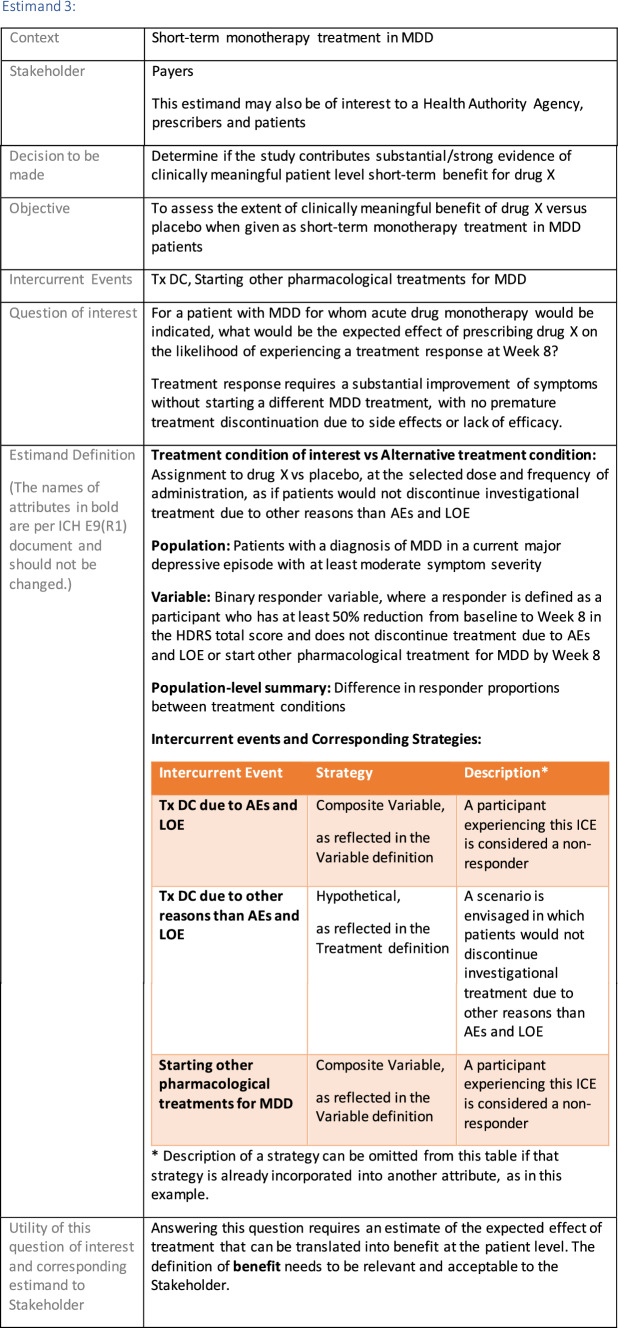
 
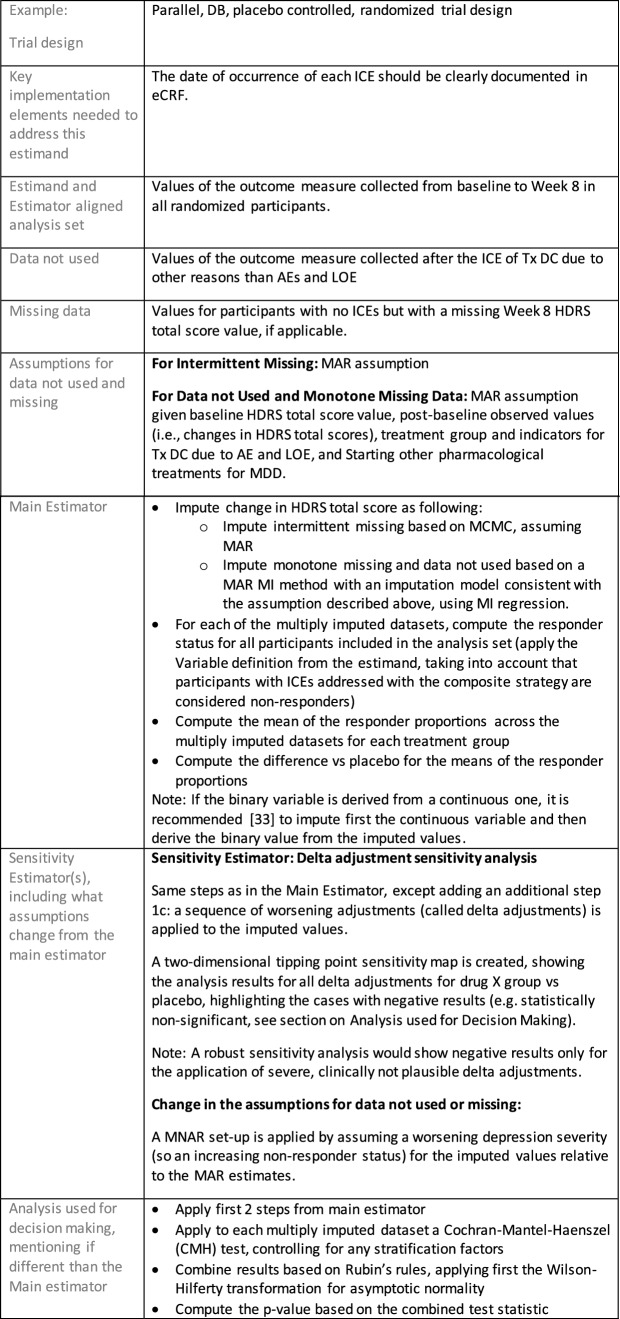



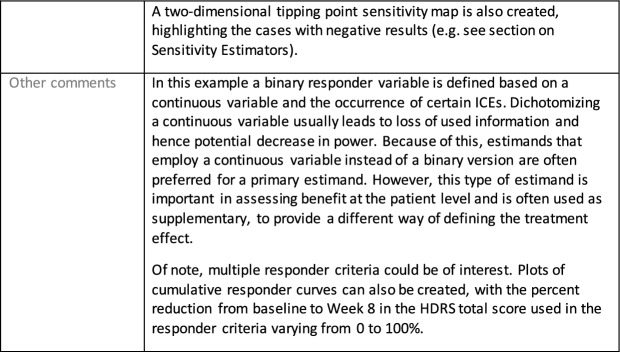
 
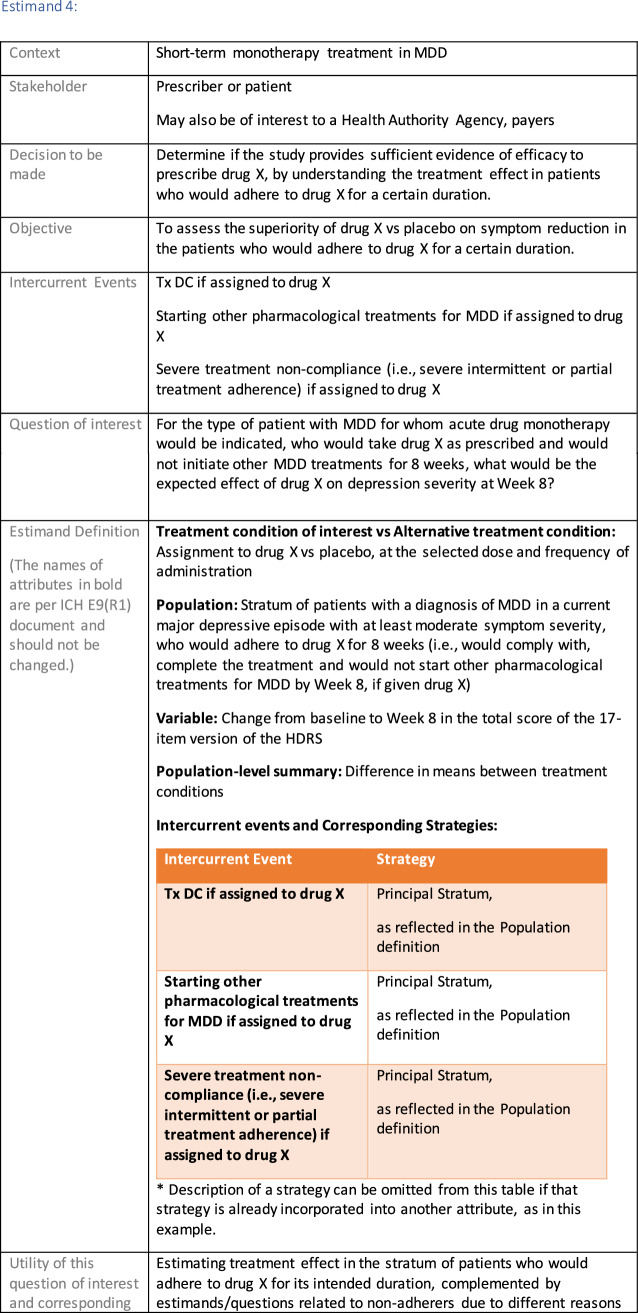
 
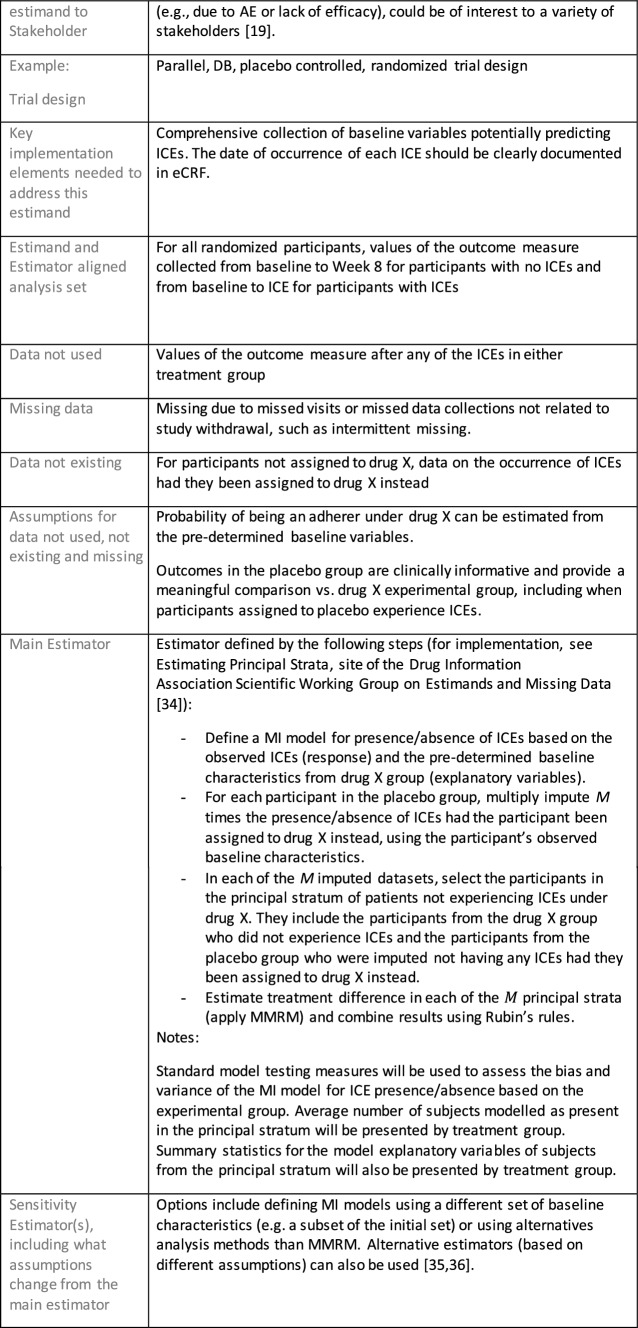



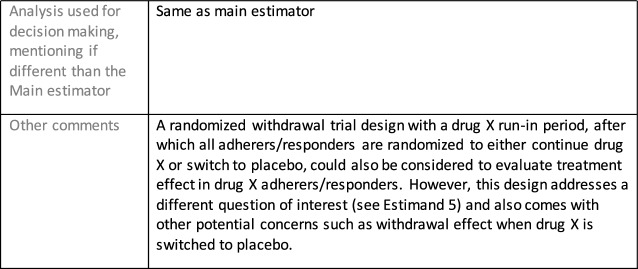
 
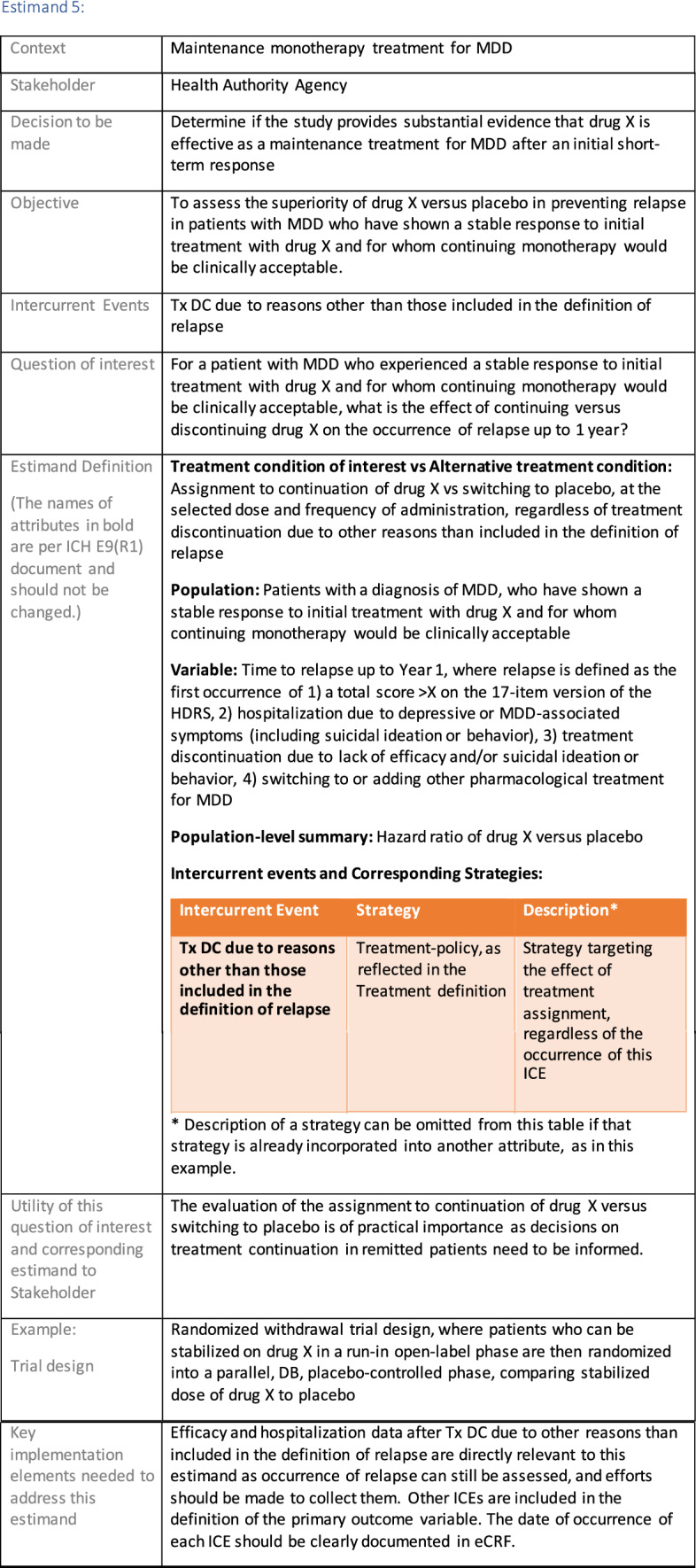
 
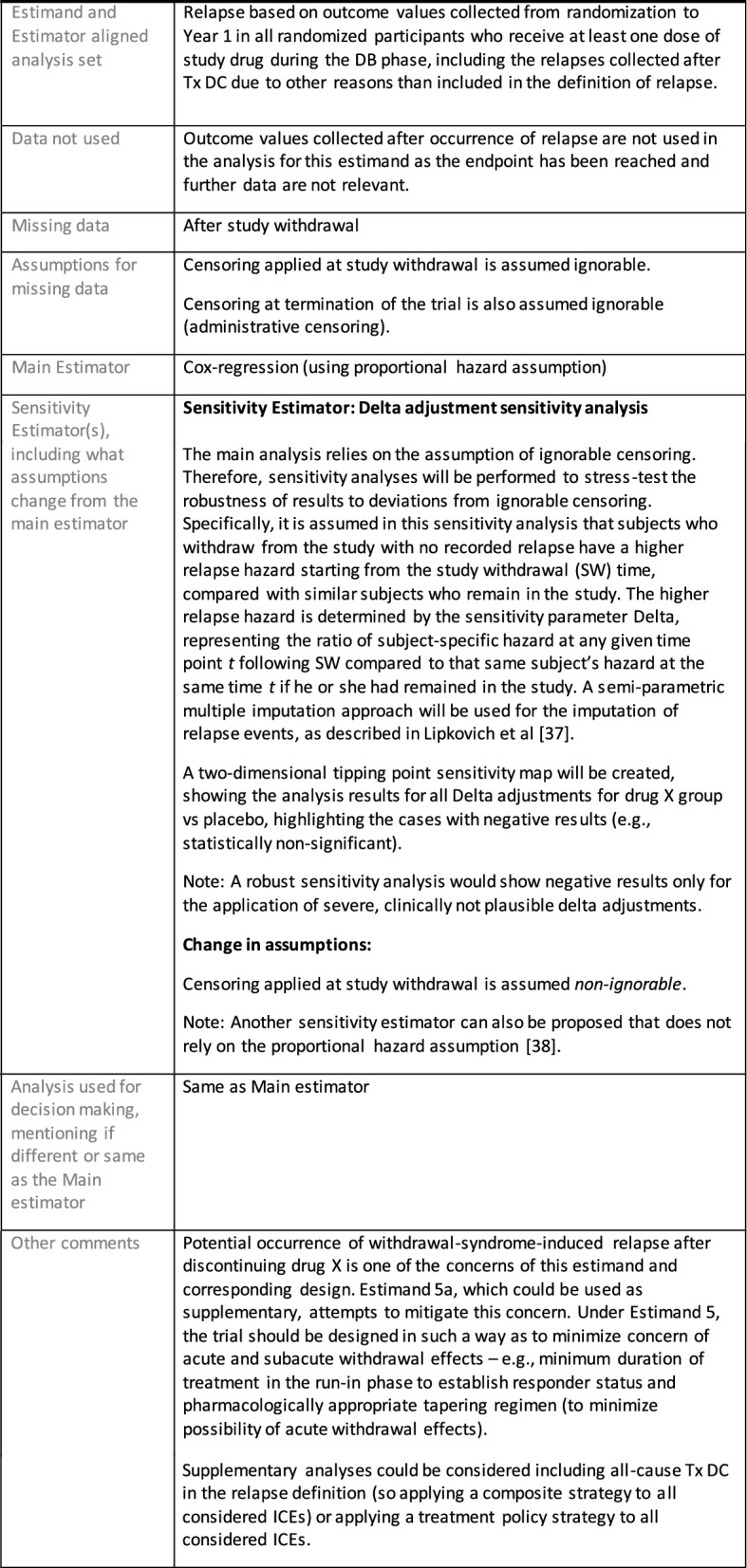



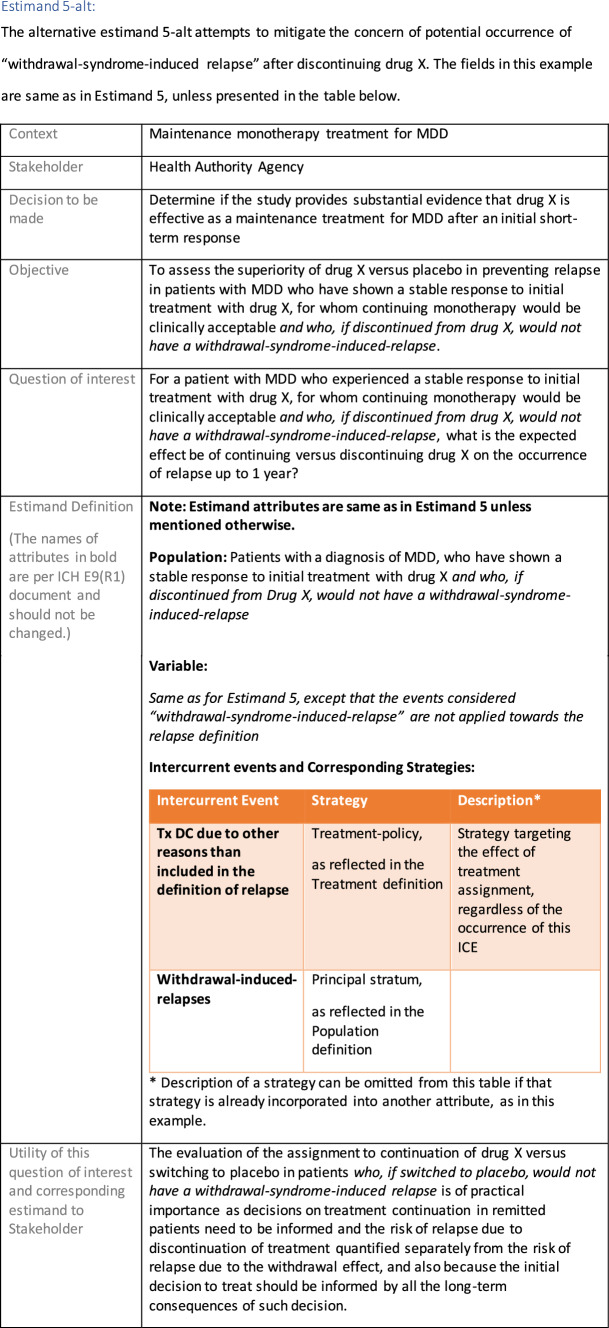
 
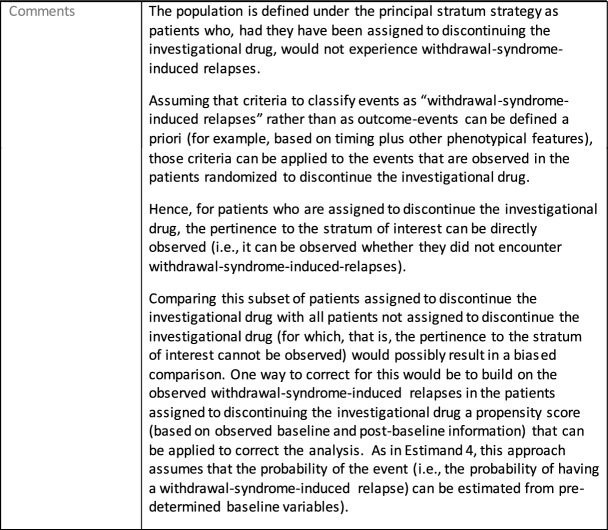



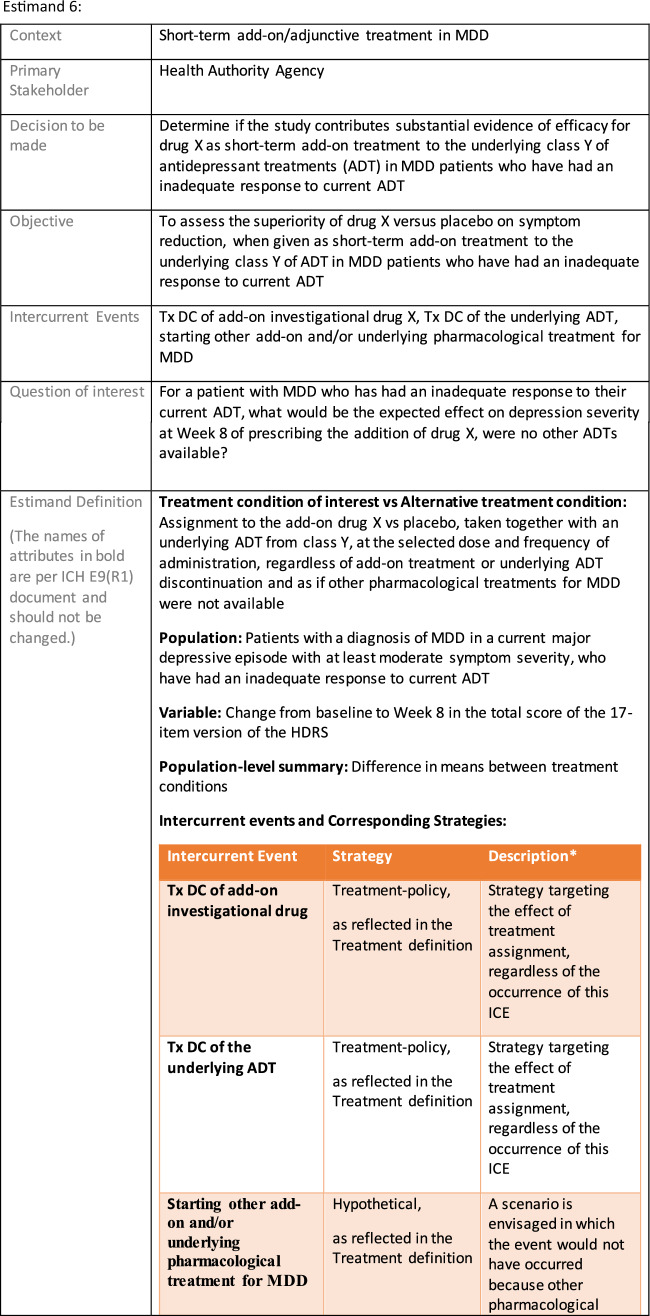
 
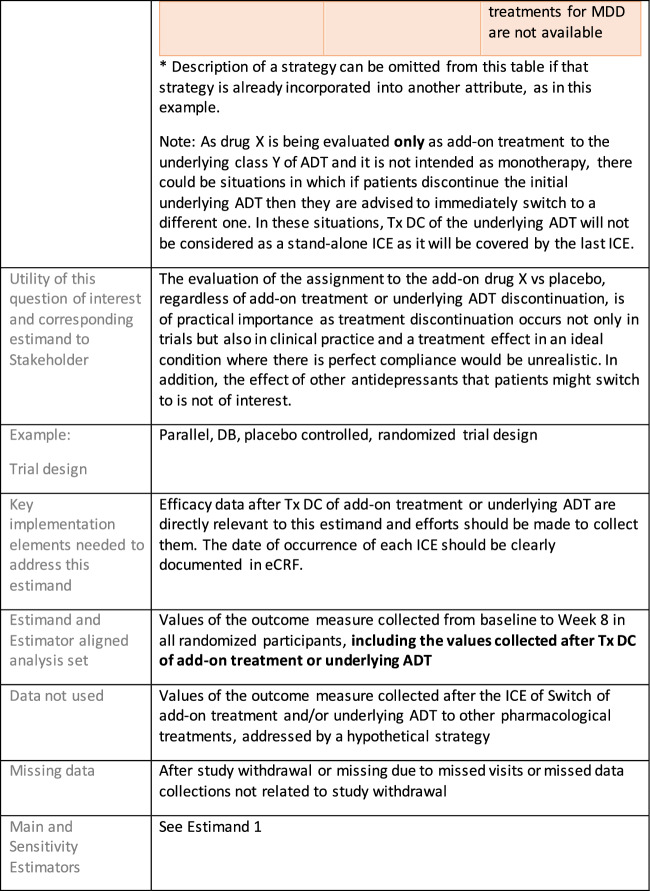


Estimands 7a and 7b:

The following estimand examples from the context of maintenance add-on/adjunctive treatment in MDD were inspired by the LQD study description from Marwood et al. [39]. They do not reflect exactly this trial original objectives and are provided as an example of estimands that complement each other. As a different example from same context, an estimand that could be aligned with the randomized withdrawal trial presented in Brunner et al. [40] could have common elements with Estimand 5 so it has not been used as an additional example for this manuscript.

Estimands 7a and 7b, defined in the following, could either be considered co-primary estimands (if the objective is to show superiority on both) or one could be considered primary and the other supplementary.
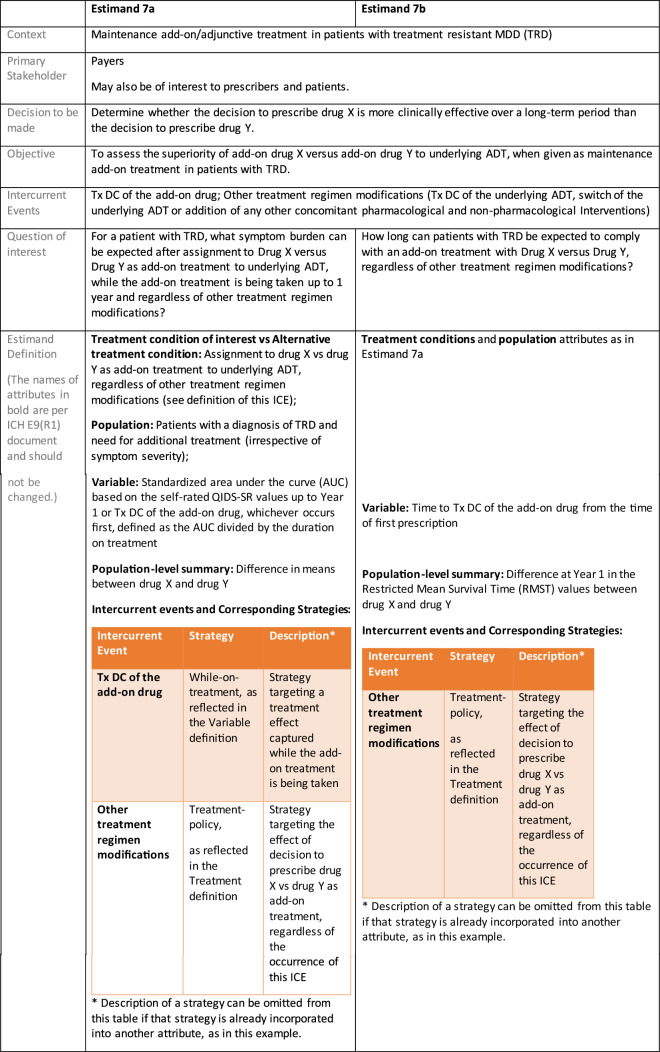

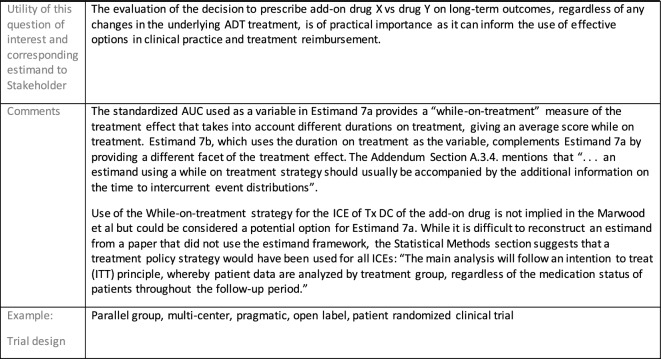


## Discussion

This paper describes an interdisciplinary process for implementing the estimand framework proposed by the ISCTM Estimand WG, a group that represents both clinical and statistical functions. Building on Bell et al. [41] and Ratitch et al. [42, 43], we expand the “thinking process” outlined in the ICH E9(R1) official training material [44] by considering the trial stakeholder(s), the decisions they need to make and the questions that would support their decision making. Study teams are encouraged to justify how answering the proposed questions of interest would support stakeholder decision-making.

The thinking process proposed is reflected in multiple examples using hypothetical trials evaluating a treatment for MDD. While this process is relevant to any therapeutic setting, all examples have been chosen to be applicable to this disease state, based on the authors’ experience.

While multiple estimand examples have been included for a given context, such as short-term monotherapy treatment in MDD, each example followed the recommended process, with clarity on the stakeholder, the decision to be made and the corresponding objective and question of interest. This is different from the previous practice (that the Addendum aims to curtail) of running multiple “sensitivity analyses”, without thought to what they estimate and their usefulness and purpose. With regard to sensitivity analyses, the Addendum recommends instead a structured approach to stress-test the assumption of the main estimator. This has been reflected in the sensitivity analyses exemplified in this paper.

In this paper we focus on the process of defining the estimand itself and do not directly address in detail the implications for the study procedures. However, the defined estimands will be reflected in the design of a study, from consent form through duration and level of follow-up to final analysis. For example, we note that selecting the estimand will lead the study team to consider logistical elements of study including.

- the burden of the study for participants (the duration of follow-up, the number of visits, complexity of data collection)
- whether to continue follow-up after an ICE (e.g., possibility of subjects remaining in the study after ICEs such as discontinuation of study treatment)
- flexibility to collect some but not all protocol assessments after treatment discontinuation or other ICE

Ultimately this paper highlights the need to incorporate multi-disciplinary collaborations into implementing the ICH E9(R1) framework and provides extensive examples on how this can be accomplished. The process described includes the element of estimand justification to foster alignment within study teams, to ensure that trials will provide answers to the most relevant clinical questions for key trial stakeholders.
